# Prognostic value of tumor growth kinetic parameters in prostate cancer patients

**DOI:** 10.18632/oncotarget.27088

**Published:** 2019-08-20

**Authors:** Gennady M. Zharinov, Oleg A. Bogomolov, Natalia Yu. Neklasova, Grigory A. Raskin, Irina V. Chepurnaya, Sergey N. Bugrov, Vladimir N. Anisimov

**Affiliations:** ^1^ Department of Radiotherapy, The Russian Research Center of Radiology and Surgical Technologies, Ministry of Health of Russian Federation, St. Petersburg, Russia; ^2^ Department of Carcinogenesis and Oncogerontology, N.N. Petrov National Medical Research Center of Oncology, Ministry of Health of Russian Federation, St. Petersburg, Russia

**Keywords:** prostate cancer, cellular lost factor, Ki-67, prostate specific antigen doubling time, prognostic factors

## Abstract

The goal of this paper was to estimate the predictive value of kinetic parameters of tumor growth in 109 prostatic cancer (PCa) patients with the morphologically verified diagnosis.

**Results:** The cell loss factor, calculated on the basis of Ki-67 values, and the PSA doubling time, proved to be an important prognostic parameter. A cumulative comparative analysis of these criteria, depending on the prevalence of the tumor process, indicates that the level of cell loss significantly decreases with increasing tumor stage (*p* = 1*10^−5^), and the growth rate of the tumor significantly increases (*p* = 1*10^−6^). In the multivariate prognostic model, the CLF is an independent predictor of tumor-specific survival along with the stage of PCa.

**Materials and methods:** For each patient of the study group were as follows. The level of Ki-67 expression in biopsies of adenocarcinoma of the prostate gland was estimated. Also, in the selected group of patients, based on the available data on the kinetics of the prostatic specific antigen (PSA), the initial time of doubling of PSA was determined. The obtained values of the actual tumor growth rate and the cell loss factor (CLF) were compared with the parameters characterizing the tumor state (stage, Gleason score, PSA level at diagnosis) and tumor-specific survival rates.

**Conclusion:** Inclusion of proliferative activity factors in nomograms and prognostic models will increase their prognostic value and practical significance. Further prospective studies are needed to analyze the actual growth rate of PCa and evaluate its proliferative activity.

## INTRODUCTION

Since the late 1950’s, growing attention was paid to study of the factors and mechanisms of tumor growth control in the humans. It was shown that the life expectancy of a cancer patient is determined by the actual rate of tumor growth. Death occurs when tumor growth reaches a “critical” volume incompatible with life [1]. It is known that in tumors there are two main opposite processes occurring simultaneously: cell division during the mitotic cycle and cell death induced by apoptosis or exposure to external causes (drug or physical exposure) [2]. The ratio of these two processes determines the growth rate of tumor mass [3]. The most common characteristic of the growth rate of tumors is the time of doubling its volume. Several methods of its calculation were proposed: flow cytometry, quantitative assessment of labeled thymidine nitrogenous bases, measurement of cellular DNA, and determination of marker expression levels [4].

The quantitative parameter of the rate of death of tumor cells from different causes is the cell loss factor (CLF). There are no direct ways to define it. CLF can be calculated in those cases when it is possible to compare the values of the actual tumor growth rate and indicators of proliferative activity of the tumor (mitotic index, Ki-67, etc.).

Evaluation of the kinetic parameters of tumor growth was carried out by many authors [5, 6, 7]. These evaluations also demonstrated the practical value of the results, namely, the prediction of radio and chemo-sensitivity of tumors (Table 1) [8].

**Table 1:** Mean kinetic parameters of various histological types of human tumors [8]

Tumor	Doubling time, days	Mitotic index, %	Cellular lost factor, %	Sensitivity to	
				Radiotherapy, Dose, Gy	Chemotherapy
Teratocarcinoma	27	90	93	25–30	++
Lymphoma	29	90	93	35–45	++
Mesenchymal sarcomas	41	11	68	85	−
Squamous cell carcinoma	58	25	89	60–70	+
Adenocarcinoma	83	6	71	60–80	±

Study of tumor growth kinetics has a real significance for clinical practice, such as evaluation of the malignant potential of each tumor and the prognosis of its growth characteristics. Due to serum prostate-specific antigen (PSA) dynamics reflecting tumor growth, prostate cancer (PCa) is a convenient model for kinetic studies on evaluation of the real rate of tumor growth [9, 10]. Parameters of the proliferative activity of PCa cells have been studied [11, 12]. However, there is no data on the interrelation of these indices with the parameters of serum PSA kinetics. Such kind of study will provide data on the meaning of relations between the proliferation and cell loss relationships.

It is well established that the clinical story of PCa widely varied in ranges from clinically insignificant, indolent, until burst-like forms. However, biomarkers and criteria, which exist in the current arsenal of oncologist, very often do not allow them to significantly predict the tumor process development [13, 14]. This stressed the need to study the pathogenesis of PCa and a search for new predictors of tumor growth.

The main goal of our study was the evaluation of the prognostic value of kinetics parameters of tumor growth in PCa patients.

## RESULTS

Mean age of patients included in the study was 66.2 ± 6.5 years. Parameters of proliferative index Ki-67 varied from 1.00% to 24.52%. The K-67 median was 10.52% (IQR 4.23–15.17%). PSADT varied from 0.27 to 232.67 months; the median was 16.83 months (IQR 1.51–40.00 months). Median of CLF was 98.2% (IQR 91.03–99.40%).

Analysis of these data has shown the following results (Tables 2–4).

**Table 2:** The values of the actual tumor growth rate and its proliferative activity depending on the prevalence of the tumor process

Tumor stage	Number of patients (%)	Median PSADT, months	p^*^	Median Ki-67, %	p^*^	Median CLF, %	p^*^
Local	42 (38.5)	39.53	1 × 10^−6^	4.50	1 × 10^−5^	99.05	1 × 10^−5^
Locally advanced	29 (26.6)	22.00		8.92		98.80	
Metastatic	38 (34.9)	1.27		15.56		90.65	

^*^Kruskal–Wallis test by ranks, Kruskal–Wallis H test.

**Table 3:** The values of the actual growth rate of the tumor and its proliferative activity depending on the initial level of PSA

Initial PSA level, ng/ml	Number of patients (%)	Median PSADT, months	p^*^	Median Ki-67, %	p^*^	Median CLF, %	p^*^
≤10.0	18 (16.5)	34.83	1 × 10^−6^	8.31	0.001	99.20	1 × 10^−6^
10.1–30.0	47 (43.1)	30.43		11.58		97.20	
30.1–100.0	28 (25.7)	2.92		12.62		91.95	
≥100.1	16 (14.7)	1.12		18.85		88.95	

^*^Kruskal–Wallis test by ranks, Kruskal–Wallis H test.

**Table 4:** The values of the actual growth rate of the tumor and its proliferative activity depending on the Gleason score

Gleason score	Number of patients (%)	Median PSADT, months	p^*^	Median Ki-67, %	p^*^	Median CLF, %	p^*^
≤6	33 (30.3)	43.33	1 × 10^−6^	4.31	1 × 10^−5^	99.20	0.0002
7	31 (28.4)	20.07		7.04		98.40	
8–10	45 (41.3)	1.67		17.62		93.50	

^*^Kruskal–Wallis test by ranks, Kruskal–Wallis H test.

Mitotic activity and Ki-67 expression in adenocarcinoma cells were increased with the increase of tumor stage, aggressiveness, and initial PSA level. As a result, a significant decrease in CLF and PSADT indices has been observed. It was revealed that there is a significant direct correlation between the rate of tumor differentiation (by Gleason score) and Ki-67 level (*r* = 0.59, *p* < 0.0001) and negative correlation with PSADT indices (*r* = −0.69, *p* < 0.0001). At the same time, there was no correlation between tumor stage and initial PSA level.

**Figure 1:**
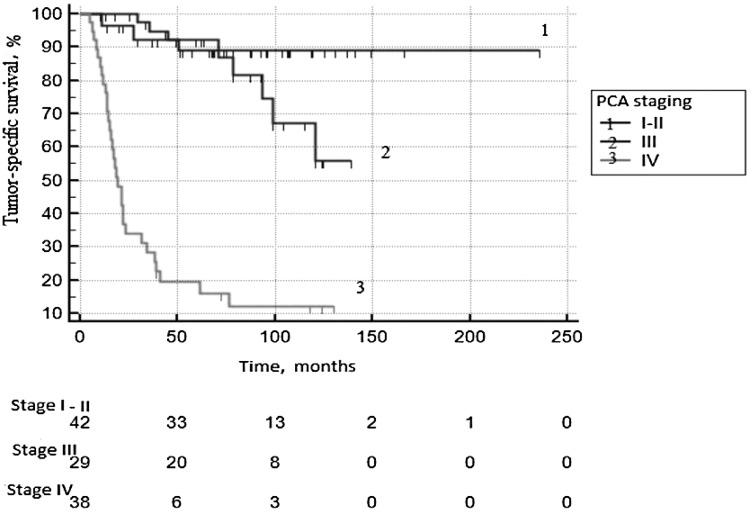
Tumor-specific survival according to PCa staging.

Tumor-specific survival (TSS) of patients of the studied group presented in Figure 1. Five years survived 89.1 ± 5.2% with local PCa; 92.4 ± 5.1% with locally advanced tumors, with metastatic cancer – 19.4 ± 6.7% (p log rank < 0.0001).

Univariate analysis showed that the PSA at diagnosis, the Gleason score, the clinical stage of PCa, the expression level of Ki-67, CLF and PSADT had a significant effect on TSS.

**Figure 2:**
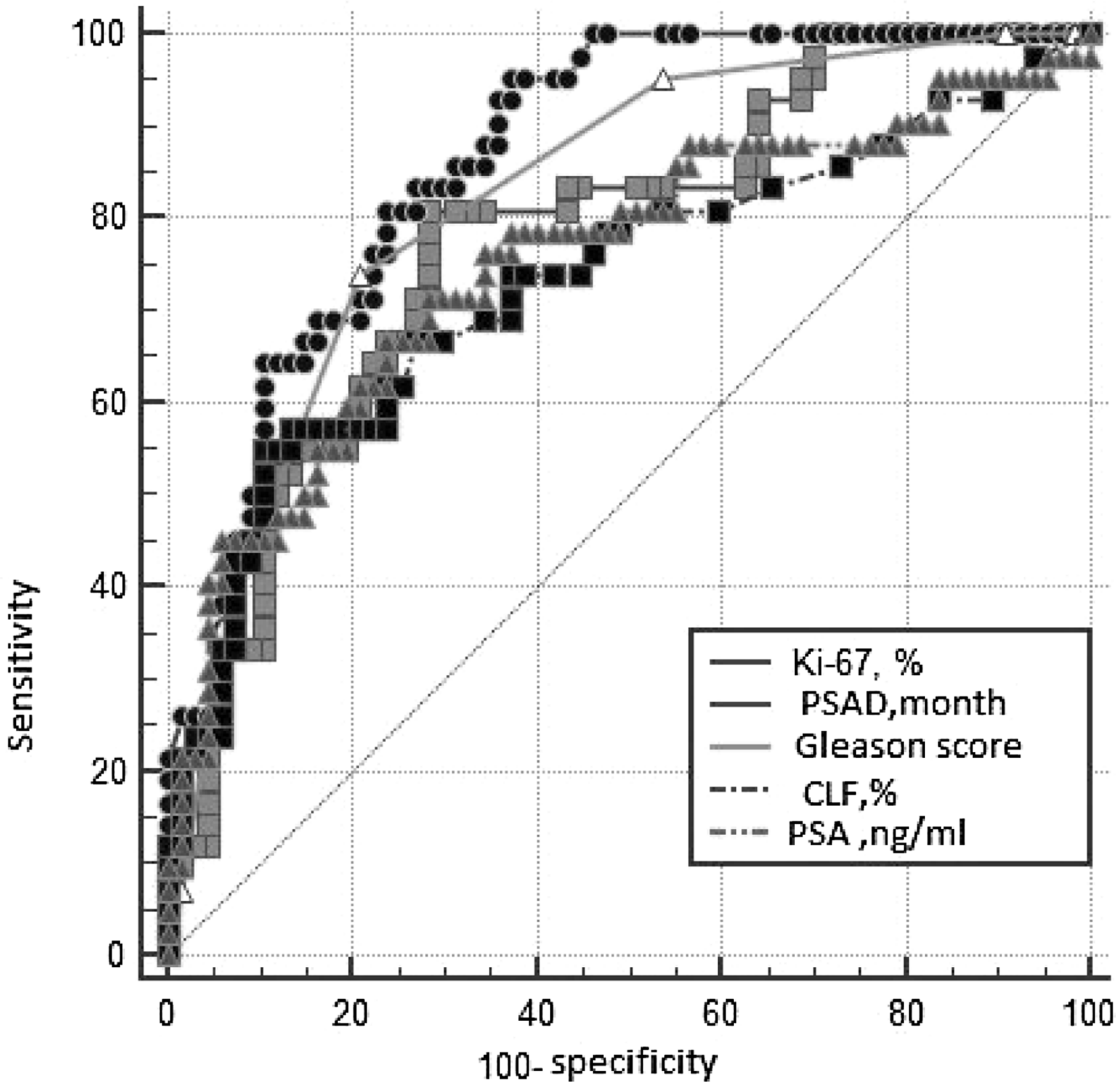
The ROC*-curves of the studied parameters. ^*^Receiver Operating Characteristic. PSAD - PSA doubling time; CLF - cell loss factor; PSA -prostatic specific antigen.

ROC analysis was performed in order to determine the predictor ability of continuous variables and calculate the threshold meaning of these parameters, subdivided all group into two subgroups significantly different by ROC parameters (Table 5; Figure 2). The analysis has shown that the optimal balance between sensitivity and specificity for the Ki-67 located at the point of 10.5%, for PSADT - 28.7 months, and for CLF - 92%. For multifactor analyses these threshold data were used for subdivision of PCa patients into prognostic subgroups: low Ki-67 (≤10.5%)/high Ki-67 (> 10.5%); low PSADT (≤28.7 months) / high PSADT (> 28.7 months); low CLF (≤92%)/high CLF (> 92%). According to the initial level of PSA 4 categories were formed: ≤10.0/10.1–30.0/30.1–100.0/≥100.1 ng/ml.

**Table 5:** Characteristics of Receiver operative curves

Parameter	AUC	SE	95% CI	*P*
Ki-67 level, %	0.779	0.0454	0.690 to 0.853	<0.001
PSADT, months	0.865	0.0334	0.786 to 0.923	
PSA, ng/ml	0.752	0.0509	0.660 to 0.829	
Gleason score	0.823	0.0386	0.738 to 0.890	
CLF, %	0.732	0.0528	0.639 to 0.812	

**Figure 3:**
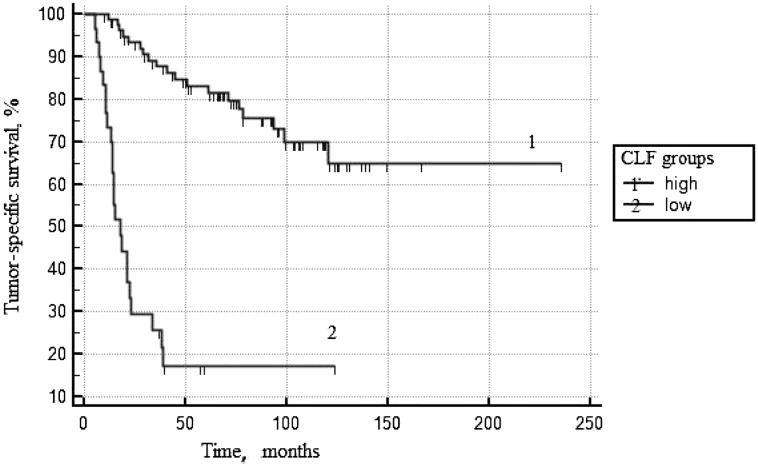
Tumor-specific survival according to CLF^*^. ^*^Cell loss factor.

Results of multifactor analyses are presented in Table 6. When clinical-morphological factors were included in the multivariant model, CLF<92% and IV stage of the disease became as independent predictors of risk of death in PCa patients. At Figure 3, the ROC curves for prognostic CLF groups are presented (*p* log rank < 0.0001).

**Table 6:** Results of multivariate analysis

Parameter	b	SE	*p*	Exp(b)	95% CI of Exp(b)
Low CLF	1.1807	0.3907	0.0025	3.2567	1.5143 to 7.0037
IV stage	1.4910	0.6563	0.0231	4.4415	1.2271 to 16.0762

## DISCUSSION

Annually, thousands of PCa patients underwent radical treatment in Russia and European countries. At the same time, it is clear that many of them could escape this therapy and the related side effects. The tactic of active observation became more common in clinical practice for this category of patients. However, used prognostic models and clinic-morphological predictors are unable in all cases to control tumor aggressiveness at various stages of the tumor process. In the era of personalized medicine, new markers which will precisely and correctly predict the natural history of PCa development and results of treatment are needed [15].

While Ki-67 is the most studied immunohistochemical marker in PCa patients, its predictor capacity in relation to parameters of survival is still under discussion. In the majority of studies, the correlation between Ki-67 level and prevalence and aggression of tumors has been reported [16, 17]. However, the results of multifactor analyzes failed to give bases for the inclusion of this parameter into prognostic nomograms due to the absence of any preferences as compared with widely used parameters, e.g., the Gleason score. Our work agrees and confirms established data on Ki-67. Increased mitotic activity and Ki-67 expression was observed in advanced aggressive prostate tumors. There is a significant direct correlation between the rate of tumor differentiation by Gleason score and Ki-67 level (*r* = 0.59, *p* < 0.0001). It was established that at the level of Ki-67 expression of more than 10.5%, the mortality risk increases by 4.5 times in PCa patients (95% CI=2.3–8.8).

PSADT is also a well-used parameter for the prognosis of the pathogenesis and results of treatment of PCa patients [9, 18]. The present work clearly shows that the rate of enhancement of serum PSA significantly correlates both with the stage of tumor process and with the rate of differentiation as evaluated by Gleason score. The PSADT parameter fits statistically significant predictor capacity in relation to tumor-specific survival in the monovariant model.

Calculated on the base of Ki-67 and PSADT parameters, the CLF indices seem the most important prognostic factors. Comparative analyses of these indices in relation to the prevalence of tumor process have shown that the level of cellular loss significantly decreases, whereas the rate of tumor growth significantly increases (*p* = 1*10^−6^). Analysis of the real rate of tumor growth and its proliferative activity in relation to the Gleason score has shown that alongside with decrease of its differentiation the rate of tumor growth also significantly increases (*p* = 1*10^−6^). However, in this case, the increase of tumor size is not only due to a decrease of cell loss (*p* = 0.0002) but also as a result of increased mitotic activity of low differentiated cells of adenocarcinoma (*p* = 1*10^−5^). In the multifactor prognostic model, CLF is an independent predictor of TSS as well as the stage of PCa. The relative risk of death at the CLF value ≤92% increases by 3.3 times. This parameter, as expected, affects in a similar direction with Ki-67, Gleason score and PSADT, and seems most valuable predictor among presented criteria.

Our observations allow including CLF in the one row with other widely used prognostic parameters. The future prospective studies to be focused upon analysis of the actual rate of growth of PCa, and its proliferative activity should be fruitful.

## MATERIALS AND METHODS

The retrospective study included 109 PCa patients who underwent hormonal and external beam radiation therapy in The A.M. Granov Russian Research Center of Radiology and Surgical Technologies from 1998 until 2015. Criteria for inclusion of patients were the presence of biopsy specimens of the prostate in the archives of the Department of Pathology of the center; a long history of blood tests for PSA (at least three within one year before the beginning of the antitumor treatment); a full set of data on outpatient examination, treatment and its results.

Table 7 depicts demographic and tumor characteristics of the study cohort.

**Table 7:** Clinical and pathologic characteristics of prostate cancer patients treated with combined hormonal and external beam radiation therapy (*n* = 109)

Age at surgery, years, M ± s	66.2 ± 6.5
BMI, Me (IQR)	25.0 (23.7–27.8)
PSA at diagnosis, ng/ml, Me (IQR)	28.6 (15.1–68.4)
Number of biopsy cores taken, Me (IQR)	6 (3–10)
Number of biopsy cores positive, Me (IQR)	4 (2–6)
Biopsy Gleason score (%)	
≤6	33 (30.3)
7	31 (28.4)
8–10	45 (41.3)
Clinical stage (%)	
local	42 (38.5)
Locally advanced	29 (26.6)
Metastatic	38 (34.9)

In all patients, the diagnosis was verified morphologically as a result of transrectal prostate biopsy. In most patients, the histological material was obtained from six sites (a minimum of three, and a maximum of 14 tissue samples). In samples of prostatic adenocarcinoma the level of expression of Ki-67 was evaluated. Estimation of Ki-67 is based on three trepan-biopsy sample whether adenocarcinoma was found in three or more biopsy patterns or on all biopsy material if adenocarcinoma was found in less than three samples. When the severe damage was detected (more than three samples) Ki-67 was researched in samples with the highest, the lowest and medium Gleason score. In order to define Ki-67 we used mouse monoclonal antibody MIB1 (DAKO) with breeding 1:50. For visualization of reaction antigen-antibody the polymer detection system EnVision Flex (DAKO company) was used (as a cromogen we took diaminobenzidine). Counterstain - enhanced banding was carried out with Mayer’s hematoxylin. The counting of Ki-67 was made by calculation of the sample mean.

All patients included in the study had at least three blood tests for PSA, performed within one year preceding the start of combination hormone-radiation therapy (maximum 12 tests). Based on the available data on the dynamics of PSA, the PSA doubling time was determined. The calculation of PSADT was carried out using an online calculator and by the accepted recommendations of the Memorial Sloan-Kettering Cancer Center, available on their website [19].

The CLF calculation was carried out according to the formula:

(1−1*Log(2)/Log(1+[Ki-67,%]/100)/[ByПCA])*100 [6].

All patients received combined hormonal and external beam radiation therapy. Calculations were made taking into account the prevalence of the tumor process and by treatment protocols adopted for the period of antitumor therapy. Patients were followed-up every three months during the first year, then every six months. The tumor-specific survival (TSS) was calculated from the time of diagnosis to the date of the last observation or death from the progression of the PCa. The objectives of the study included assessing the prognostic significance of the Ki-67 and PSADT levels for the survival of patients with PCa, as well as determining the correlation dependence of the parameters and the characteristics of the tumor process. The obtained values of the actual growth rate of the tumor and CLF were compared with the parameters characterizing the tumor status (stage, Gleason score, initial PSA level). The CLF value in the prognosis of the survival of patients with PCa was also evaluated.

For statistical analysis, MedCalc 14.12.0 (MedCalc Software, Belgium) was used. The mean (M) and the standard deviation (s) were used to characterize interval variables having a normal distribution, for the characterization of ordinal and interval variables not subject to a normal distribution - the median (Me) and the interquartile range (IQR) The differences between groups with normal distribution were assessed using Student’s *t*-test. The differences between the two groups in the absence of approximately normal distribution were assessed using the Mann-Whitney *U* test. The Kruskal-Wallis test was used for comparing more than two independent samples. The relationship between qualitative characteristics was evaluated by using the Pearson Chi-square test and risk assessment.

If necessary, to determine the threshold values of interval variables that divide the sample into groups that are statistically significantly different in survival, the ROC (receiver operative curve) analysis method was used. Pairwise comparison was completed using Kaplan-Meier curves with the log-rank test. A multivariable proportional hazards Cox regression model was used to determine factors associated with progression-free survival. The criterion for statistical reliability of the findings was the level of significance *p* < 0.05.
